# First Example of a Lipophilic Porphyrin-Cardanol Hybrid Embedded in a Cardanol-Based Micellar Nanodispersion

**DOI:** 10.3390/molecules171012252

**Published:** 2012-10-18

**Authors:** Ermelinda Bloise, Luigi Carbone, Giuseppe Colafemmina, Lucia D’Accolti, Selma Elaine Mazzetto, Giuseppe Vasapollo, Giuseppe Mele

**Affiliations:** 1 Department of Engineering for Innovation, University of Salento, via Arnesano, 73100 Lecce, Italy; Email: ermelinda.bloise@unisalento.it (E.B.); giuseppe.vasapollo@unisalento.it (G.V.); 2 National Nanotechnology Laboratory (NNL), Institute of Nanoscience CNR, Via Arnesano 16, 73100 Lecce, Italy; Email: luigi.carbone@nano.cnr.it; 3 Chemistry Department, University of Bari “A. Moro”, via Orabona, 4, 70126 Bari, Italy; Email: colafemmina@chimica.uniba.it (G.C.); lucia.daccolti@uniba.it (L.D.); 4 Laboratory of Products and Processes Technology (LPT), Department of Organic and Inorganic Chemistry, Federal University of Ceará, Fortaleza 6021, Brazil; Email: selma@ufc.br

**Keywords:** cardanol, cholesterol, lipophilic porphyrin, cardanol hybrid, micellar dispersion

## Abstract

Cardanol is a natural and renewable organic raw material obtained as the major chemical component by vacuum distillation of cashew nut shell liquid. In this work a new sustainable procedure for producing cardanol-based micellar nanodispersions having an embedded lipophilic porphyrin itself peripherally functionalized with cardanol substituents (porphyrin-cardanol hybrid) has been described for the first time. In particular, cardanol acts as the solvent of the cardanol hybrid porphyrin and cholesterol as well as being the main component of the nanodispersions. In this way a “green” micellar nanodispersion, in which a high percentage of the micellar system is derived from renewable “functional” molecules, has been produced.

## 1. Introduction

**Figure 1:**
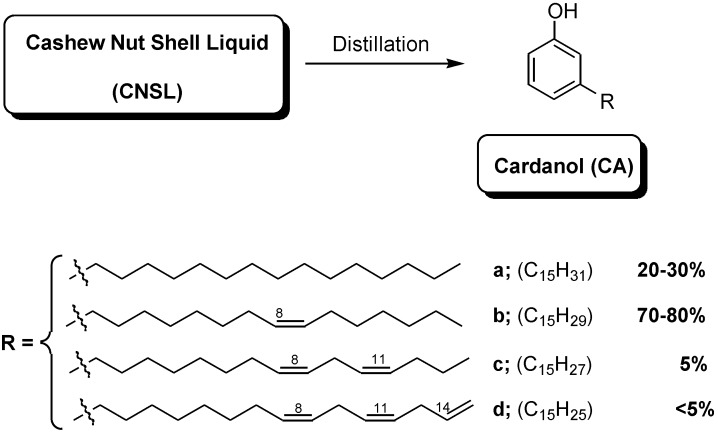
Molecular structure and composition of cardanol (CA).

Cardanol (CA) is a natural and renewable organic raw material obtained as a major component by vacuum distillation of cashew nut shell liquid (CNSL) [1,2]. The chemistry of CA is becoming a stimulating area in academic and industrial research, particularly for the preparation of new eco-friendly fine chemicals, composites and functional organic materials [3]. Indeed CA, is a mixture of 3-*n*-pentadecylphenol (20–30%), 3-(pentadeca-8-enyl)phenol (70–80%), 3-(penta-deca-8,11-dienyl)- phenol (nearly 5%), and 3-(pentadeca-8,11,14-trienyl) phenol (less than 5%) (Figure 1).

Over the last few years CA has attracted scientists for its potential use in resins, friction lining materials, surface coatings, as an antioxidant, [4] in organic synthesis, nanomaterials [5,6,7,8,9] and molecular hybrid systems [10,11,12,13]. It was also recently used as a natural product to develop hybrid systems containing macrocycles such as porphyrins [14,15,16] which are considered to be very attractive compounds due to their extensive application in many areas of new materials such as chemical technology, ecology, medicine, electronics and photocatalytic processes [17,18,19,20,21].

Peculiar properties of CA and its derivatives, such as the relatively high solubility in non-polar environments and good processability, can be ascribed to the presence of the -C_15_- chain attached to the *meta* position of the phenolic ring.

Some resorcinolic lipids, like cardol and 2-methylcardol present in minor amount in CNSL, are especially receiving increasing attention as potential candidates in drug delivery systems (DDS) because of their amphiphilic properties promoting the formation of liposomal structures under alkaline conditions [22]. Stability and permeability of the phospholipid bilayer of 1-palmitoyl-2-oleylphosphatydilcholine liposomes was demonstrated using CA as an easily available non-toxic replacement for cholesterol in drug targeting [23].

The development of vesicular nanosystems doped with selected porphyrins [24] and metalloporphyrins has given rise to an extensive search in the medical field. Electron spin resonance studies focused on metalloporphyrins intercalated in liposome membranes [25] have highlighted the principal reasons why in a lipid bilayer its initial well organized setup may be considerably rearranged by metalloporphyrins that permeate through it. The resulting arrangement of membrane components depends on stereochemical properties as steric hindrance, molecular structure flexibility, and electrostatic interactions arising from distribution of electron density in the metal’s coordination sphere. Hence, any disruption within the well-organized lipid structure of a cell membrane may considerably affect the vital function of the cell itself. Controlled membrane modification leading to its presumed dysfunction may be interesting in tumor treatment, especially as diverse porphyrins and their derivatives have been shown to accumulate selectively in cancer cells [26].

Additionally, cationic metalloporphyrins, synthesized as superoxide dismutase mimics, were introduced into niosomes and then demonstrated to be highly effective in promoting O_2_^−^**^.^** radical decomposition and employed as DDS in antioxidant drugs’ preparation [27,28].

## 2. Results and Discussion

**Figure 2:**
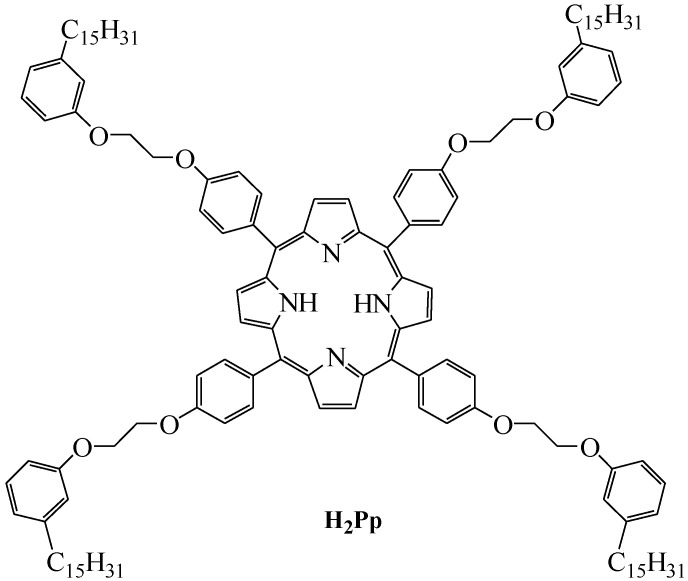
Structure of the lipophilic porphyrin-cardanol derivative (H_2_Pp).

With the aim of obtaining a natural lipid-based system from renewable organic raw materials, our study was undertaken starting from the above-mentioned researches with a special emphasis on the ability of CA to form a stable vesicle dispersion when mixed with cholesterol (CH) under alkaline conditions [22], as well as on the expected capability of a lipophilic porphyrin-cardanol hybrid (H_2_Pp) [14], shown in the Figure 2, to be intercalated into the hydrophobic layer of the vesicle structure.

A colloidal nanodispersion, hereafter generally referred to as micellar dispersion, has been prepared according to the thin film hydration method [29,30]. This technique is usually used to prepare vesicle dispersions employing volatile organic solvents to dissolve lipid components.

**Scheme 1:**
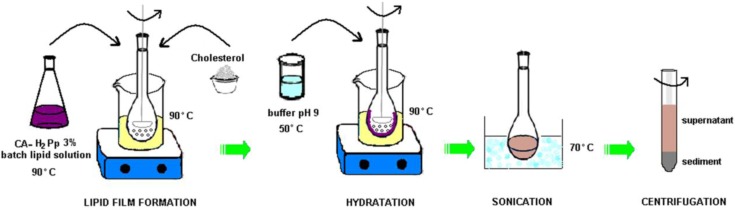
Details of the nanodispersion preparation.

**Figure 3:**
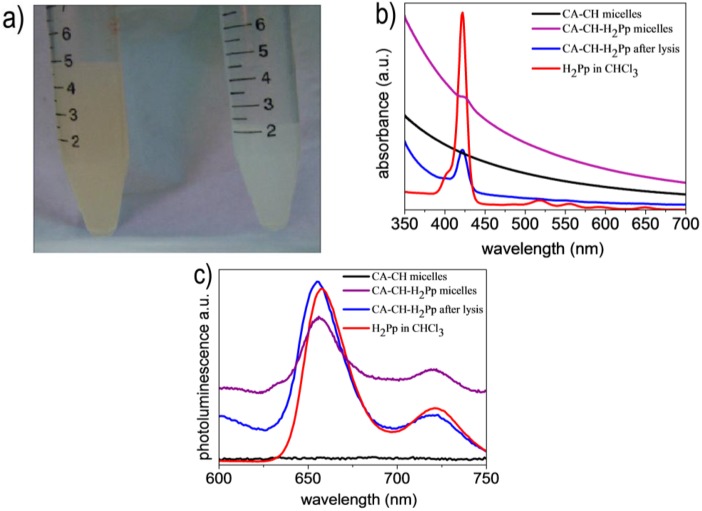
(**a**) Comparison of a CA-CH-H_2_Pp (left side) micellar nanodispersion in buffer solution with a CA-CH (right side) blank sample. (**b**) UV–visible absorption and (**c**) photoluminescence spectra respectively of CA-CH micelles in buffer solution (black line), CA-CH-H_2_Pp micelles in buffer solution (purple line), CA-CH-H_2_Pp micelles after their lysis and solubilisation in chloroform (blue line) and H_2_Pp hybrid solution in chloroform (red line).

In this work we propose a new sustainable way of producing cardanol-based micellar nanodispersions in which CA acts as solvent of the cardanol hybrid H_2_Pp and CH and as well as being the main component of the micellar dispersions. Scheme 1 summarizes the main steps of sample preparation. Typically, a 3% solution of H_2_Pp in CA is mixed with CH (molar ratio of CA:CH is 1:0.85) then homogenized by stirring in a round bottom flask, under mild heating, until a homogeneous lipidic film is formed. The hydration step producing the micellar nanodispersion consists in the addition of a warm buffer solution to the lipidic film: an alkaline environment is a critical condition for micelle formation. The presence of small glass beads in the reaction batch enhances the surface contact between oil and water phases and promotes formation of the micelles. It is important to note that CH molecules added in the formulation increase the solubilization capability of emulsion and therefore act as co-surfactant, so that samples prepared with only CA, without CH, do not form spherical encapsulating micelles, and instead a clear water and oil phase separation occurs. A sonication step, subsequent to the initial hydration step, has an important bearing on best micellar size distribution, while a centrifugation procedure favors the separation of smallest micelles (CA-CH-H_2_Pp) in supernatant that appears as an emulsion ivory coloured (Figure 3a, left).

A blank sample (CA-CH) has been prepared by mixing CA oil with CH at the previously reported molar ratio as described above, until a homogeneous milky white emulsion is formed. The first feature giving evidence that the lipophilic porphyrin-cardanol hybrid is embedded in the hydrophobic core of micelles is the colour difference between CA-CH-H_2_Pp sample and blank reference (Figure 3a). This has been further confirmed by comparison of UV-visible absorption and photoluminescence spectra.

As shown in Figure 3b, the blank sample CA-CH does not exhibit any characteristic absorption throughout the investigated spectral region, merely showing an increasing optical density at lower wavelengths. On the contrary, the spectra of CA-CH-H_2_Pp micelles in buffer solution and even further after their lysis and solubilisation in chloroform display a net absorption at 423 nm corresponding to the Soret band typical of cardanol-porphyrin derivative in chloroform [14].

Analogously, the shape of photoluminescence spectra of CA-CH-H_2_Pp micelles both in buffer solution and after their lysis, is very similar to the emission spectra of H_2_Pp measured in chloroform (Figure 3c).

The micelle hydrodynamic diameter has been determined by dynamic light scattering (DLS). The hydrodynamic diameter is defined as the diameter that a set of identical spheres should have in order to diffuse light at the same rate of the particles being measured.

**Figure 4:**
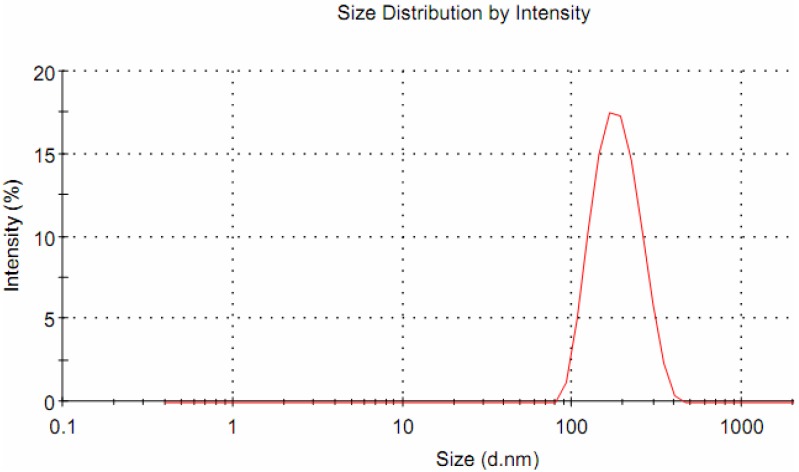
Size distribution of CA-CH-H_2_Pp micellar nanodispersion obtained by DLS measurements.

Figure 4 shows the light scattering curve of CA-CH-H_2_Pp as a function of particle diameter which results rather size monodispersed, with a mean hydrodynamic diameter of 179 nm (PDI = 0.077) and a Z-potential value of −72.0 mV. Similar results have been obtained for the blank reference CA-CH thus indicating that the size of micelle is not affected by the embedded H_2_Pp.

**Figure 5:**
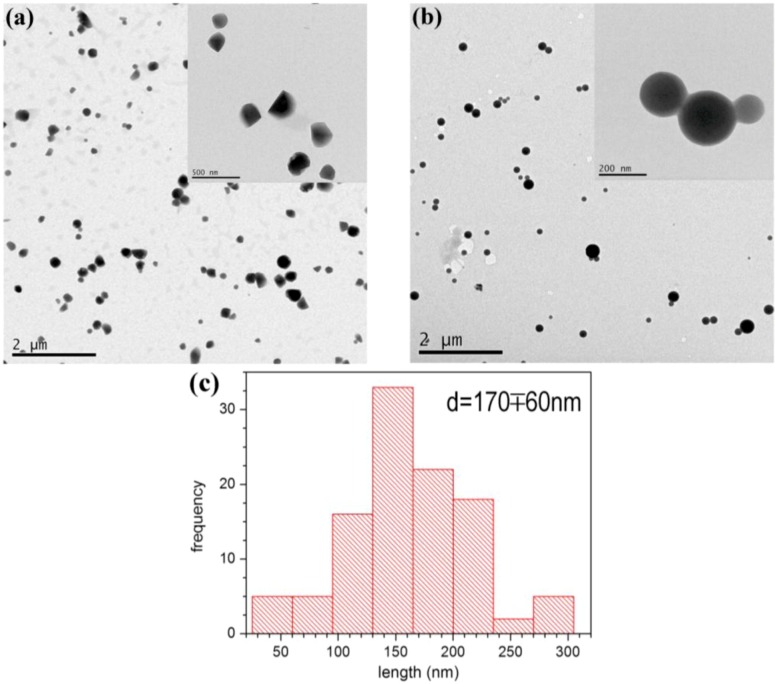
Transmission electron micrographs of (**a**) CA-CH (blank) and (**b**) CA-CH-H_2_Pp micellar nanodispersions stained with osmium tetroxide aqueous solution for enhancing the contrast. Insets in the pictures report magnified areas of each sample. (**c**) Particle-size distribution of sample CA-CH-H_2_Pp.

Figure 5 reports TEM microphotographs respectively of CA-CH and CA-CH-H_2_Pp micellar dispersions, in which a reshaping occurring in the presence of H_2_Pp is highlighted. Whether from one side the blank sample shows irregular architectures, on the other side the CA-CH-H_2_Pp solution exhibits perfectly spherical profiles and homogeneity in the size distribution. 

As shown in Figure 5b, the porphyrin H_2_Pp has an influence on the micellar shape distribution. The observed regular spherical shape could be ascribed to the interactions deriving from the intercalation of the lipophilic side chains of H_2_Pp with the micellar hydrophobic core.

^1^H-NMR analysis confirmed the presence of all components (CA, CH and H_2_Pp) both in the supernatant as well as in the sediment. In particular, the spectra registered after separation, drying and lysis of the micelles exhibited typical signals of the porphyrin moiety (8.89 ppm, protons at the β position of the pyrrole unit), the signals of the aromatic protons of cardanol and the aromatic rings of the porphyrin (8.20–6.70 ppm range) and typical multiplets of the methyl groups of cholesterol. 

Moreover, the ^1^H-NMR analysis performed on the sediment deriving from blank sample (CA-CH) and CA-CH-H_2_Pp sample, after 60 days stored at room temperature, confirmed the presence of all the unassembled components. These results proved that the composition of the micelles don’t change despite their very slow tendency to form aggregates.

Besides supplementary and more detailed analysis of self-diffusion coefficients of the water peak, using the NMR technique, shown that the *D_water_* is 2.1 × 10^−9^ m^2^s^−1^ is closely to value of the coefficient of the neat water (2.3 × 10^−9^ m^2^s^−1^); [31] this means that the water is free to move and the aggregates were quite similar to vesicles.

## 3. Experimental

Cholesterol, KCl, H_3_BO_4_, NaOH were purchased from Sigma-Aldrich (Steinheim, Germany) and used as received; methanol and chloroform used in analysis routes were HPLC grade; ultra pure (UP) water delivered by a Zeneer UP 900 Human Corporation system. Borate buffer solution pH 9.0 is a UP water solution of H_3_BO_4_ 30 mM, KCl 70 mM and NaOH 18 mM. Porphyrin-cardanol based (H_2_Pp), prepared according with the procedure reported in [14], was added to cardanol oil (CA) under stirring and then heated (90 °C) in order to prepare a batch lipidic solution of CA/H_2_Pp 3% (w/w). Preliminary tests have shown that 3% H_2_Pp represents an appropriate amount to obtain a complete solubilization in cardanol oil.

Lipidic solution (33 mg), cholesterol (36 mg) and glass beads (5 g, diameter = 4 mm) were mixed by mechanical stirring at 90 °C for 1 h to form a lipidic film on the flask’s wall. The resulting film has been hydrated with 20 mL of borate buffer pH 9.0 preheated at 50 °C under mechanical stirring (700 rpm) and, finally, heated at 90 °C for 1 h. The as-obtained micellar dispersion was submitted to a sonication step (45 min at 70 °C) and then centrifuged (7,000 rpm for 30 min) thus collecting the supernatant as sample. The sediment residue was separated and dried at 60 °C to constant weight. The difference in weight provides the yield of supernatant sample as 45% of organic components. A sample without H_2_Pp has been prepared with the same procedure as blank reference.

The morphology of the micellar dispersion was examined using transmission electron microscopy (TEM). Low-magnification TEM analyses were performed on a Jeol JEM-1011 electron microscope operating at 100 kV, equipped with a CCD camera ORIUS 831 from Gatan. TEM samples were prepared by initially mixing dilute micellar dispersions with a few microliters of 1% (w/v) osmium tetroxide aqueous solution and then drop-casting them onto carbon-coated copper grids. Hence each grid is twice rinsed in pure water and afterwards, the deposited samples are completely dried at 60 °C for one night before examination.

^1^H-NMR spectra were recorded on a Bruker Avance 400 at room temperature. The micelles in supernatant (5 mL) have been lysed in methanol (10 mL), then dried and dissolved in CDCl_3_. The sediment, which is separated after 60 days from the supernatant stored at room temperature, has been washed with UP water, dried and then dissolved in CDCl_3_. ^1^H-NMR (25 °C, TMS): H_2_Pp [6] δ, ppm 8.89 (s, 8H, β position of the pyrrole moiety), 8.15 (d, *J* = 8.6 Hz, 8H, Ar), 7.36 (d, *J* = 8.6 Hz, 8H, Ar), 7.30 (t, *J* = 7.8 Hz, 4H, Ar), 6.96-6.82 (m, 12H, Ar), 4.72–4.50 (m, 16H, OCH_2_), 2.67 (t, *J* = 7.7 Hz, 8H, Ar-CH_2_), 1.72–1.60 (m, 8H, Ar-CH_2_-CH_2_), 1.39–1.26 (m, 96 H, CH_2_), 0.89 (t, *J* = 7.0 Hz, 12H, CH_3_), −2.73 (br s, 2H, NH); CA δ, ppm 0.97 (t, -(CH_2_)_n_-CH_3_, 3H), 1.37–1.44 (m, -CH_2_-, 16H), 1.64 (m, ArCH_2_-CH_2_-, 2H), 2.08 (m, -CH_2_-CH=CH-CH_2_-, 4H), 2.60 (t, *J* = 7.4 Hz, Ar-CH_2_-, 2H), 2.85 (m, -CH=CH-CH_2_-CH=CH-, 1H), 5.43 (m, -CH=CH-, 2H), 6.70 (d, Ar, 1H), 6.72 (s, Ar, 1H), 6.80 (d, Ar, 1H), 7.18 (t, *J* = 7.6 Ar, 1H); CH δ, ppm 5.35 (dd, *J* = 7.3, =CH-CH_2_-, 1H), 0.85 (d, *J* = 6.6, 3H, CH_3_), 0.87 (d, *J* = 6.6, 3H, CH_3_).

Self-diffusion coefficients measurements have been carried out by the Fourier transform NMR pulsed field gradient spin-echo (PFGSE-NMR) method [32] using VARIAN Inova400 spectrometer operating at 400 MHz. The pulse sequence employed was a Stejskal-Tanner sequence [31] 90°-ô-180°-ô-echo with two rectangular field gradient pulses of 8 ms, separated by a constant interval D = 50 ms. The echo amplitude is given by *I = I_o_ × exp*[*−γ*^2^g^2^δ^2^(Δ−δ/3)D]were γ is the gyromagnetic ratio of proton, D is the self-diffusion coefficient of the species responsible of the spin-echo decay and g ranged from 1 to 18 G^.^cm^−1^. The strength of the applied field gradient (G) was determined by a separate calibration with pure H_2_O.

Measurement of Dynamic Light Scattering and Electrophoretic Light Scattering were both carried out on a Malvern Zetasizer Nano ZS on diluted samples, to establish the size and zeta potential of micelles. The hydrodynamic diameter of micellar dispersion has been determined at 25 °C measuring the autocorrelation function at 90° scattering angle. Cells have been filled with 400 µL of sample solution and diluted to 4 mL with UP water. Five separate measurements have been made to derive average.

The presence of porphyrin-cardanol hybrid embedded into a micellar dispersion has been evidenced by UV–visible and photoluminescence measurements carried out by using Varian Cary 300 and Varian Cary Eclipse spectrophotometers. The measurements were carried out by direct analysis both of the opportunely diluted micellar dispersion (ratio 1:2 v/v of micellar dispersion:buffer) and of the mixture of free molecular components after lysis obtained by dissolving 1 mL of colloidal solution in 5 mL of methanol then allowing the solvent to evaporate at 70 °C under vacuum and finally dissolving the residue in 2 mL of chloroform.

## 4. Conclusions

In this work a “green” nanodispersion, in which a high percentage of the system is derived from renewable “functional” molecules, has been produced. For the first time a new sustainable procedure of producing cardanol-based micellar nanodispersions having an embedded lipophilic porphyrin peripherally functionalized with cardanol substituents (porphyrin-cardanol hybrid) arranged in a vescicular structure has been proposed.
